# Geographic Distribution of Environmental Relative Moldiness Index Molds in USA Homes

**DOI:** 10.1155/2011/242457

**Published:** 2011-06-07

**Authors:** Stephen Vesper, Jennie Wakefield, Peter Ashley, David Cox, Gary Dewalt, Warren Friedman

**Affiliations:** ^1^National Exposure Research Laboratory, United States Environmental Protection Agency, Cincinnati, OH 45268, USA; ^2^Consolidated Safety Services, Dynamac Corporation, Cincinnati, OH 45268, USA; ^3^U.S. Department of Housing and Urban Development, Washington, DC 20410, USA; ^4^Environmental Sciences Group QuanTech, Arlington, VA 22201, USA

## Abstract

*Objective*. The objective of this study was to quantify and describe the distribution of the 36 molds that make up the Environmental Relative Moldiness Index (ERMI). *Materials and Methods*. As part of the 2006 American Healthy Homes Survey, settled dust samples were analyzed by mold-specific quantitative PCR (MSQPCR) for the 36 ERMI molds. Each species' geographical distribution pattern was examined individually, followed by partitioning analysis in order to identify spatially meaningful patterns. For mapping, the 36 mold populations were divided into disjoint clusters on the basis of their standardized concentrations, and First Principal Component (FPC) scores were computed. *Results and Conclusions*. The partitioning analyses failed to uncover a valid partitioning that yielded compact, well-separated partitions with systematic spatial distributions, either on global or local criteria. Disjoint variable clustering resulted in seven mold clusters. The 36 molds and ERMI values themselves were found to be heterogeneously distributed across the United States of America (USA).

## 1. Introduction

Some attempts have been made to describe the geographic distribution of molds in United States of America (USA) homes and buildings. Horner et al. [1] quantified culturable molds from air and dust samples obtained from 50 single family residences in Atlanta, GA, USA. Shelton et al. [2] reported on the analysis of culturable indoor and outdoor air samples from 1,717 buildings in the USA. Nearly 50% of the buildings were located in the “southeast” USA. These samples came from inspectors as part of their investigations of these buildings. Thus, these buildings did not represent a random sampling. 

 Protocols for indoor mold population studies have never been standardized. Therefore, it is virtually impossible to compare one study with another. US EPA and HUD researchers recently standardized the analysis of molds in US housing based on settled dust [3]. As part of the 2006 HUD American Healthy Home Survey (AHHS), standardized dust samples were obtained from a statistically representative set of homes across the USA. Each of these dust samples was prepared in the same way and then analyzed using mold-specific quantitative PCR (MSQPCR) [3]. The 36 molds include the 26 Group 1 species which we have shown to be associated with homes with water damage and the 10 Group 2 species which are found in homes independent of water damage [4, 5]. If the Sum of the Logs of the Group 2 (SLG2) molds is subtracted from the Sum of the Logs of the Group 1 (SLG1) molds, a unitless Environmental Relative Moldiness Index (ERMI) value is obtained which describes the mold burden in a home with a single numeric value relative to a National ERMI scale [3]. The ERMI scale ranges from about −10 to 20 or even higher and is divided into quartiles [3]. The development of the ERMI, its components and calculations, has recently been reviewed [6]. 

 The analysis of molds in dust by MSQPCR has been used by us to estimate the mold burden in homes, defined by both the concentrations of the molds as well as the diversity of species in the home [4, 5, 7, 8]. By using a standard protocol for sampling homes and a DNA-based method of analysis, the distribution of the 36 molds that make up the Environmental Relative Moldiness Index (ERMI) can be described for the USA.

## 2. Materials and Methods

### 2.1. Home Selection Process

The American Healthy Homes Survey (AHHS) targeted a nationally representative sample of permanently occupied homes or housing units. A housing unit is defined as a house, apartment, mobile home, a group of rooms, or a single room that is occupied as separate living quarters. Separate living quarters are those in which the occupants live and eat separately from any other persons in the building and which have direct access from the outside or through a common hall.

 For this survey, lists of households in the sampled segments were acquired from commercially available sources. A sample of four residential addresses, plus two backup addresses, was randomly selected from the list in a typical segment to determine which households were eligible to be included in the sample. These lists were validated by a modified listing process in which interviewers visited the sampled segments with the acquired lists to compare them with the housing units actually present to validate a list for each designated segment within each “primary sampling unit” (PSU). This comparison resulted in some housing units being added to the lists and others being deleted from the lists [3]. Because of low population density, no samples were obtained from the states of Oregon, Montana, Nevada, Utah, Wyoming, North Dakota, or South Dakota.

### 2.2. Dust Sample Collection and Analysis

Dust samples were collected by vacuuming 2 m^2^ of the living room floor and 2 m^2^ of a bedroom floor (whether carpeted or not), directly adjacent to the sofa or bed, respectively, for 5 min each with a Mitest sampler-fitted vacuum. The analysis of 5 mg of sieved dust from each sample was completed by EPA licensed commercial laboratories, as previously described [3]. All primer and probe sequences, as well as known species comprising the assay cluster and the list of EPA licensed commercial laboratories, are published at the EPA website: http://www.epa.gov/microbes/moldtech.htm. 

### 2.3. Statistical Methodology

The AHHS set of data included concentrations of each of the 36 molds for each of the 1083 homes in the continental US. Home locations were converted to latitude and longitude coordinates prior to data exploration and reduction.

 The data were initially inspected for evidence of geographical influence on mold burden using regression analysis on the 1083 home data set. Separate regressions were run for each species and ERMI-related index (SLG1, SLG2, and ERMI) using a maximum likelihood regression procedure with log10-transformed mold concentrations and nontransformed ERMI indices as the response variable and latitude and longitude as predictors.

 Data were then assessed for the presence of distinct spatial partitions of homes on the basis of standardized concentration values for the 36 mold species. Spatially mediated partitions were generated using the *clustTool* [9] package for R [10], with multiple combinations of distance metric, partitioning algorithm and number of partitions being tested and compared to obtain optimal partitioning.

After the partitioning, data reduction was carried out in two separate stages in order to create a manageable visual representation based on a small number of relatively homogeneous mold groups by clustering together those species with similar patterns of abundance as a function of geographic distribution. Once molds were assigned to clusters, the abundance measures for each of the molds in a cluster were combined to form a single cluster component, preferentially weighted on those molds with greatest information content. This reduction step was carried out using the SAS 9.2 VARCLUS procedure (SAS Inst., Inc., Cary, NC), resulting in a first principal component (FPC) for each cluster that, for mapping purposes, was representative of concentrations and geographical distributions of the subset of molds belonging to that cluster.

 The second data reduction step prerequisite to mapping was carried out in order to collapse FPC scores across “sampling locales”, that is, across groups of geographically-related homes that were sampled as representatives of a metropolitan population along the lines of the original PSUs. This step served to provide a local estimate of the mold burden in the community being sampled. Sampling clusters were identified on the basis of a disjoint cluster analysis carried out on latitude and longitude data using the SAS FASTCLUS procedure, which reduced the number of plotting positions from 1083 to 82. The UNIVARIATE procedure was used to calculate the mean latitude and longitude of each of the 82 sampling locales for plotting purposes, as well as mean FPC scores and ERMI-related indices for each locale.

 First Principal Component scores and ERMI-related values were plotted by latitude and longitude as an *x*, *y* event-layer using the software program ArcGIS Desktop 9.3.1 (ESRI Inc., Redlands, CA). For each map, the values were classified using a five category natural break classification. The five categories were indicated by combined grey scale and graduated symbol size, with the smallest size and lightest color representing the category with the smallest values.

## 3. Results and Discussion

### 3.1. Results

The initial regression analyses suggest that for at least some of the 36 mold species, a significant, systematic relationship exists between the concentration of the mold and the geographic gradient (Table 1). Thirteen of the 26 Group 1 species and 8 of the 10 Group 2 species fall into this category, as do the indices Sum of the Logs of Group 2 and ERMI. The nature of these relationships, however, was inconsistent even among the species within Groups 1 or 2: Positive longitude coefficients coupled with negative latitude coefficients are found for species in both Groups 1 and 2 molds, as are negative longitude coefficients coupled with positive latitude coefficients (Table 1). 

 The partitioning analyses based on the full set of 1083 homes and 36 mold species failed to uncover a valid partitioning that yielded compact, well-separated partitions with systematic spatial distributions, either on global or local criteria (data not shown). Irrespective of variations in distance metric, partitioning algorithm or predefined number of partitions, validity measures remained relatively stable within a very small range, inconsistent with the presence of true partitions that become increasingly distinct as the optimal number of partitions is reached: average silhouette widths, for example, were consistently low, most on the order of 0.05–0.07, and very few reaching as high as 0.1 (data not shown).

 These results, combined with those from individual mold species, suggest that while some species' concentrations may generally increase/decrease to some (variable) extent along a preferred geographical gradient, the individual trends do not intersect to form patterns that represent coherent species communities in association with spatially distinct subsets of individual homes. Thus species concentrations, when seen as an integrated multispecies system, are heterogeneously distributed across the continental US. Given the apparent absence of statistically valid partitions of this type, we focused instead on results from the data reduction techniques employed for mapping purposes and provide a more broad scope description of those findings in light of the single-species results.

 The variable clustering analysis of the 36 molds' standardized concentrations produced seven disjoint clusters at criterion, as shown in Table 2. The R^2^ values associated with each mold listed in Table 2 represent the squared correlations between the molds themselves and, respectively, the cluster to which they were assigned and the next closest cluster. A large R^2^ value for a given mold with its own cluster indicated a better fit of the mold data to the cluster component and was in turn associated with greater weighting of that mold in the calculation of FPC scores.

**Figure 1:**
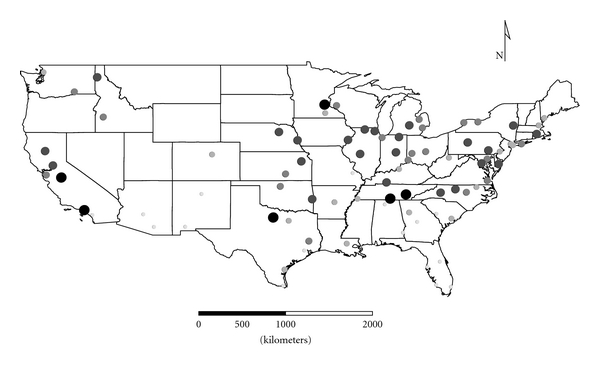
The First Principal Component (FPC) scores derived from the molds in Cluster 1 (*Aspergillus ochraceus, A. penicillioides, A. restrictus, A. sclerotiorum, A. versicolor, Scopulariopsis chartarum, *and *Wallemia sebi*) plotted by latitude and longitude as an *x*, *y* event layer using the software program ArcGIS Desktop 9.3.1 (ESRI Inc., Redlands, CA). The values were classified using a five-category natural break classification. The five categories are indicated by combined grey scale and graduated symbol size, with the smallest size and lightest color representing the category with the smallest values.

**Figure 2:**
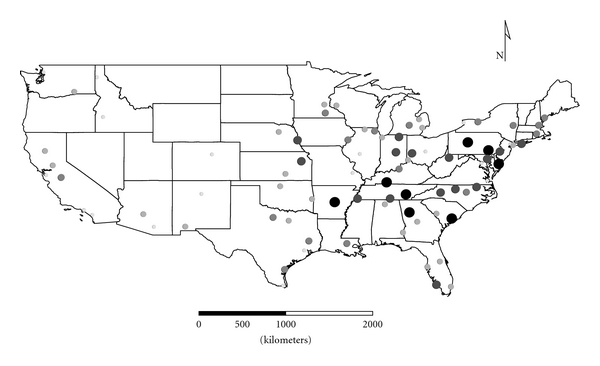
The First Principal Component (FPC) scores derived from the molds in Cluster 2 (*Chaetomium globosum*, *Cladosporium sphaerospermum*,* Penicillium *Group 2, and *Trichoderma viride*) plotted by latitude and longitude as an *x*, *y* event layer using the software program ArcGIS Desktop 9.3.1 (ESRI Inc., Redlands, CA). The values were classified using a five-category natural break classification. The five categories are indicated by combined grey scale and graduated symbol size, with the smallest size and lightest color representing the category with the smallest values.

**Figure 3:**
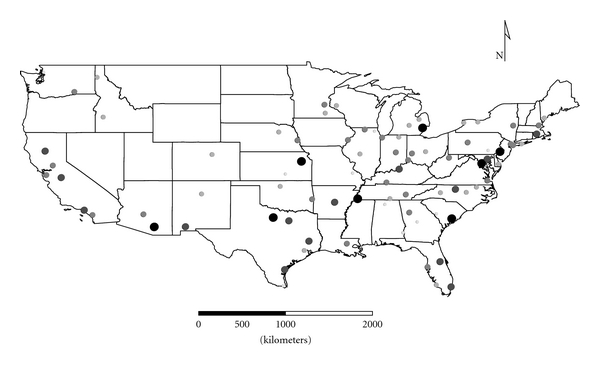
The First Principal Component (FPC) scores derived from the molds in Cluster 3 (*Aspergillus fumigatus*, *Paecilomyces variotii*, *Penicillium brevicompactum*, *P. corylophilum,* and *P. variabile*) plotted by latitude and longitude as an *x*, *y* event layer using the software program ArcGIS Desktop 9.3.1 (ESRI Inc., Redlands, CA). The values were classified using a five category natural break classification. The five-categories are indicated by combined grey scale and graduated symbol size, with the smallest size and lightest color representing the category with the smallest values.

**Figure 4:**
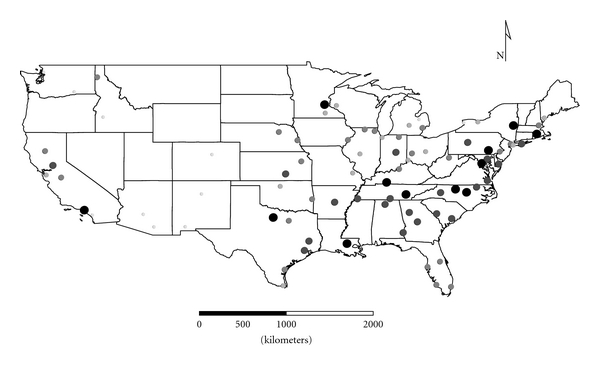
The First Principal Component (FPC) scores derived from the mold in Cluster 4 (*Penicillium purpurogenum*) plotted by latitude and longitude as an *x*, *y* event layer using the software program ArcGIS Desktop 9.3.1 (ESRI Inc., Redlands, CA). The values were classified using a five-category natural break classification. The five categories are indicated by combined grey scale and graduated symbol size, with the smallest size and lightest color representing the category with the smallest values.

 Clusters 1 to 4 were exclusively Group 1 molds. Cluster 1 was dominated by *Aspergillus,* and the distribution appeared strongest in the eastern half of the country (Figure 1), as expected given the positive longitude coefficients associated with Cluster 1 and lack of significant negative longitude coefficients. Cluster 2 FPC scores had a wide distribution (Figure 2) except in the desert southwest. Consistent with the mix of positive and negative coefficients, Cluster 3 FPC scores were distributed more widely in the western US (Figure 3) than were scores from some of the other exclusively Group 1 Clusters. Cluster 4 was specifically *Penicillium purpurogenum*, concentrations of which did not pattern significantly with either longitude or latitude, and it was found as expected randomly distributed across the entire US (Figure 4). 

**Figure 5:**
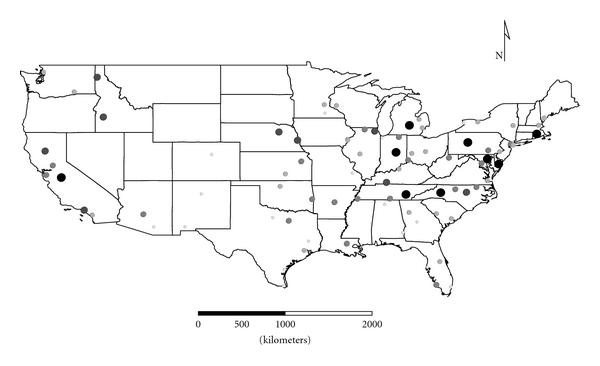
The First Principal Component (FPC) scores derived from the molds in Cluster 5 (*Acremonium strictum*, *Alternaria alternata*,* Cladosporium cladosporioides*, *C. herbarum,* and *Epicoccum nigrum*) plotted by latitude and longitude as an *x*, *y* event layer using the software program ArcGIS Desktop 9.3.1 (ESRI Inc., Redlands, CA). The values were classified using a five-category natural break classification. The five categories are indicated by combined grey scale and graduated symbol size, with the smallest size and lightest color representing the category with the smallest values.

**Figure 6:**
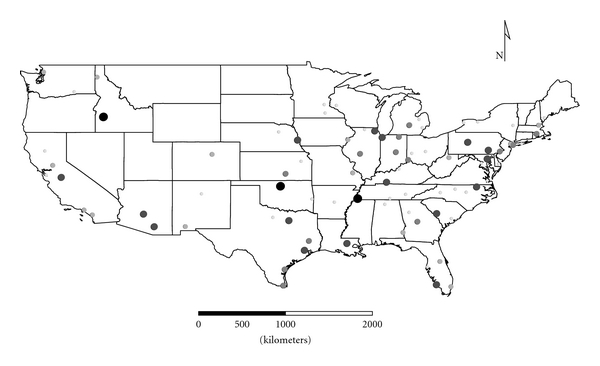
The First Principal Component (FPC) scores derived from the molds in Cluster 6 (*Aspergillus flavus*, *A. niger*, *A. sydowii*, *A. unguis*, *Penicillium spinulosum*, *Aspergillu ustus,* and *Penicillium chrysogenum* Type 2) plotted by latitude and longitude as an *x*, *y* event layer using the software program ArcGIS Desktop 9.3.1 (ESRI Inc., Redlands, CA). The values were classified using a five-category natural break classification. The five categories are indicated by combined grey scale and graduated symbol size, with the smallest size and lightest color representing the category with the smallest values.

**Figure 7:**
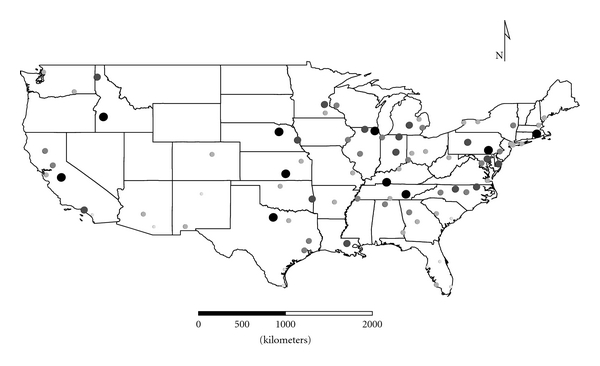
The First Principal Component (FPC) scores derived from the molds in Cluster 7 (*Aureobasidium pullulans*, *Eurotium amstelodami, Scopulariopsis brevicaulis*, *Stachybotrys chartarum*, *Mucor racemosus, *and* Rhizopus stolonifer*) plotted by latitude and longitude as an *x*, *y* event layer using the software program ArcGIS Desktop 9.3.1 (ESRI Inc., Redlands, CA). The values were classified using a five-category natural break classification. The five categories are indicated by combined grey scale and graduated symbol size, with the smallest size and lightest color representing the category with the smallest values.

 Cluster 5 was made up exclusively of Group 2 molds (6 of the 10 Group 2 molds), 4 of which were significantly associated with geographical gradients (Figure 5). Clusters 6 (Figure 6) and 7 (Figure 7) were mixtures of Group 1 and 2 molds.

**Figure 8:**
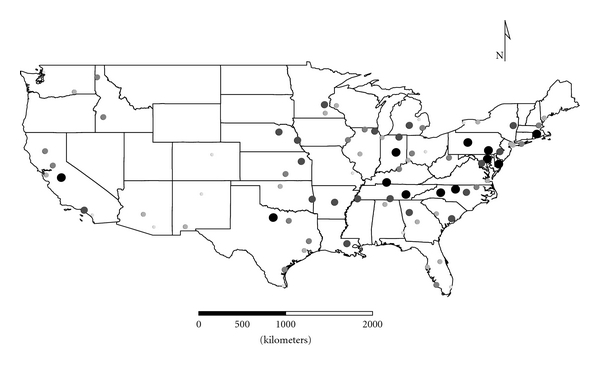
The Sum Logs Group 1 values plotted by latitude and longitude as an *x*, *y* event layer using the software program ArcGIS Desktop 9.3.1 (ESRI Inc., Redlands, CA). For each map, the values were classified using a five-category natural break classification. The five categories are indicated by combined grey scale and graduated symbol size, with the smallest size and lightest color representing the category with the smallest values.

**Figure 9:**
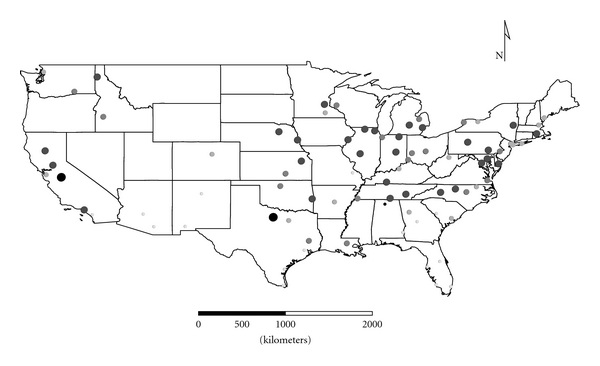
The Sum Logs Group 2 values plotted by latitude and longitude as an *x*, *y* event layer using the software program ArcGIS Desktop 9.3.1 (ESRI Inc., Redlands, CA). The values were classified using a five-category natural break classification. The five categories are indicated by combined grey scale and graduated symbol size, with the smallest size and lightest color representing the category with the smallest values.

**Figure 10:**
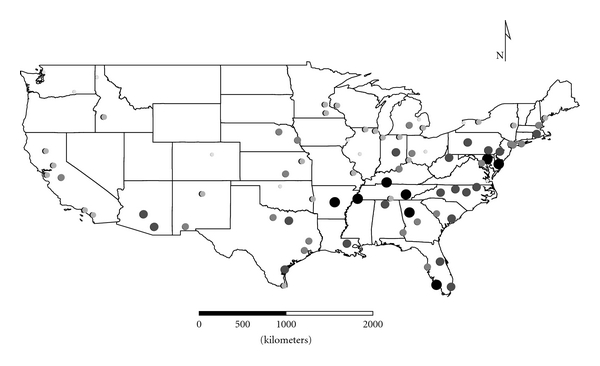
The ERMI values plotted by latitude and longitude as an *x*, *y* event layer using the software program ArcGIS Desktop 9.3.1 (ESRI Inc., Redlands, CA). The values were classified using a five-category natural break classification. The five categories are indicated by combined grey scale and graduated symbol size, with the smallest size and lightest color representing the category with the smallest values.

 The distribution of the Sum of the Logs of the Group 1 (SLG1) molds (Figure 8) showed a mix of high and low values scattered across the USA. The distribution of the Sum of the Logs of the Group 2 (SLG2) molds (Figure 9) was fairly uniform across the USA with most locales in the darker shades of gray. The ERMI values themselves were heterogeneously distributed across the continental US (Figure 10).

### 3.2. Discussion

This representative national survey of US homes is the first attempt to apply a standardized sampling procedure and DNA-based quantification of molds in settled dust to describe the geographic distribution of specific molds. The metric for understanding the mold burden in homes is the ERMI scale which is made up of 36 molds, 26 indicators of water damage and 10 outdoor molds [3]. This survey demonstrates that these 36 molds have a national distribution. In fact, these 36 molds have been shown to also occur from the UK [10] to Singapore [11]. 

 Humidity and precipitation are the natural phenomena that can alter the moisture conditions in a home but it is primarily the homes' internal and external structural integrity that controls moisture and mold growth. Therefore, the SLG1 molds (Figure 8) will vary not only by geography but based on natural and man-made moisture conditions in each home, dominated by the latter. The result is Group 1 mold populations being “speckled” across the USA as in Figure 8, with a mix of high and low values indicating that water problems are national in scope. 

 On the other hand Group 2 molds accumulate as a function of outdoor conditions (soil, vegetation, etc.) and the habits of the home's occupants, for example, how often do they clean, do they leave the windows open, are there pets and so forth. Most areas of the USA showed a fairly uniform distribution of Group 2 molds with few extremes (Figure 9). Only in the desert southwest and the southeast does it appear that Group 2 molds are less abundant than in the rest of the USA. Desert outdoor conditions in the southwest or the subtropical southeast conditions may distinguish these home outdoor mold populations to some extent. 

 The ERMI calculation (subtracting SLG2 from SLG1) adjusts for these variations in order to provide a relative scale for comparison of homes. The resulting ERMI values demonstrate the heterogeneous distribution of mold in homes across the USA (Figure 10). Of course, this is a simplification, and any particular house may or may not support the growth of any of these molds. So the origins of a particular mold population could be a mixture of both inside and outside sources [6]. However, in general Group 1 and Group 2 molds segregated into separate clusters. This suggests that their population sources are largely separate, that is, Group 1 from indoors (Figures 1 to 4) and Group 2 (Figure 5) from outdoors.

 The cooccurrence of certain species in the FPC Clusters demonstrates that specific molds tend to colonize homes in common patterns. For example, *Aspergillus,* dominating Cluster 1, appears to populate homes in the eastern USA and *Penicillium,* dominating Cluster 3, populates homes in the western USA. Perhaps these differences are a function of different types of building practices, age of housing, and so forth. More intense investigation of these locale differences may help explain specific health issues associated with molds [12]. 

 Past surveys of mold concentrations have relied on air samples. For example, Shelton et al. [2] reported on the analysis of over 9,000 indoor air samples and 2,400 outdoor air samples from 1717 buildings. They found that *Cladosporium*, *Penicillium*, nonsporulating fungi, and *Aspergillus* were the most common molds. However, these samples were not collected at random but came from buildings with employee health complaints, in the evaluation of visible mold growth or odors, or from a “proactive” indoor air quality program. So there was a bias in the sampling locations. Also, the samples were short-term air samples, and these types of samples have many inherent limitations [6, 13–16]. 

 The overall goal of our mold research is to place mold analysis on a firmer, more objective basis. However, any methodology also has to be practical. Of the possible hundreds of molds in a home, only monitoring 36 is a limitation but the results described here suggest that these 36 molds are nationally distributed.

## 4. Conclusion

The ERMI values in homes were found to be widely and heterogeneously distributed across the USA indicating that the 36 molds that make up the ERMI are broadly distributed with only limited geographic selection.

## Figures and Tables

**Table 1:** Assessment of relationships between mold species concentrations or ERMI-related indices and the longitude and latitude coordinates of the 1083 survey homes. *Z* values represent regression coefficients. Those values that are significant are italicized.

Molds	Longitude		Latitude	
	*Z* value	*P* value	*Z* value	*P* value
Group 1				
* Aspergillus flavus*	−*2.374 *	*0.018*	1.437	0.151
* Aspergillus fumigatus*	−0.985	0.325	1.476	0.140
* Aspergillus niger*	−*5.152 *	*<0.001*	*4.537*	*<0.001*
* Aspergillus ochraceus*	−1.392	0.164	*2.127*	* 0.033*
* Aspergillus penicillioides*	1.019	0.308	0.163	0.871
* Aspergillus restrictus*	1.705	0.088	−1.271	0.204
* Aspergillus sclerotiorum*	1.530	0.126	−1.522	0.128
* Aspergillus sydowii*	0.499	0.618	−1.266	0.205
* Aspergillus unguis*	0.067	0.946	−0.341	0.733
* Aspergillus versicolor*	*2.801*	0.005	−2.165	*0.030*
* Aureobasidium pullulans*	−*3.217 *	0.001	3.662	*<0.001*
* Chaetomium globosum*	1.243	0.214	1.087	0.277
* Cladosporium sphaerospermum*	0.852	0.394	−0.663	0.507
* Eurotium *group	−*3.332 *	*<0.001*	*4.836*	* <0.001*
* Paecilomyces variotii*	0.629	0.529	−0.232	0.817
* Penicillium brevicompactum*	−*2.970 *	*0.003*	*3.289*	* 0.001*
* Penicillium corylophilum*	−*2.104 *	*0.035*	*2.751*	* 0.006*
* Penicillium crustosum*	−1.303	0.193	1.734	0.083
* Penicillium purpurogenum*	−0.044	0.965	−0.408	0.683
* Penicillium spinulosum*	−1.607	0.108	1.695	0.090
* Penicillium variabile*	*3.012*	*0.003*	*−2.72*	* 0.006*
* Scopulariopsis brevicaulis*	−0.450	0.653	1.019	0.308
* Scopulariopsis chartarum*	−1.835	0.067	*2.647*	* 0.008*
* Stachybotrys chartarum*	*−3.296*	*<0.001*	*3.192*	* 0.001*
* Trichoderma viride*	*3.636*	*<0.001*	*−3.413*	*<0.001*
* Wallemia sebi*	*4.215*	*<0.001*	*−3.720*	*<0.001*

Group 2				
* Acremonium strictum*	−0.168	0.866	*2.108*	*<0.001*
* Alternaria alternata*	*−8.768*	*<0.001*	*9.706*	*<0.001*
* Aspergillus ustus*	*−2.878*	*0.004*	*2.451*	* 0.014*
* Cladosporium cladosporioides *1	−0.472	0.637	1.952	0.051
* Cladosporium cladosporioides *2	*−8.110*	<0.001	9.179	<0.001
* Cladosporium herbarum*	*−6.994*	<0.001	8.957	<0.001
* Epicoccum nigrum*	0.510	0.610	1.504	0.133
* Mucor *group	*4.648*	*<0.001*	*−3.985*	*<0.001*
* Penicillium chrysogenum *2	*−3.456*	*<0.001*	*3.935*	*<0.001*
* Rhizopus stolonifer*	*−2.743*	*0.006*	*2.659*	* 0.008*

ERMI Indices				

Sum Logs Group 1	−0.372	0.710	1.067	0.286
Sum Logs Group 2	*−3.878*	*<0.001*	*5.599*	*<0.001*
ERMI	*2.538 *	*0.011*	*−2.953*	* 0.003*

**Table 2:** The SAS 9.2 VARCLUS procedure (SAS Inst., Inc., Cary, NC) was applied to standardized log data to divide the 36 species into disjoint clusters such that each species belonged to one and only one internally homogeneous cluster. All species began in a single cluster, which was split iteratively to maximize the variance accounted for by the cluster components. Splitting continued until the eigenvalue associated with each cluster's second principal component reached the criterion of a maximum of 1.0.

Cluster		ERMI group	*R* ^2^ own cluster	*R* ^2^ next closest
(1)	*Aspergillus ochraceus*	1	0.340	0.096
	*Aspergillus penicillioides*	1	0.475	0.166
	*Aspergillus restrictus*	1	0.317	0.034
	*Aspergillus sclerotiorum*	1	0.353	0.048
	*Aspergillus versicolor*	1	0.379	0.098
	*Scopulariopsis chartarum*	1	0.361	0.129
	*Wallemia sebi*	1	0.509	0.141

(2)	*Chaetomium globosum*	1	0.514	0.148
	*Cladosporium sphaerospermum*	1	0.535	0.211
	*Penicillium *Group 2	1	0.198	0.053
	*Trichoderma viride*	1	0.478	0.116

(3)	*Aspergillus fumigatus*	1	0.325	0.133
	*Paecilomyces variotii*	1	0.474	0.102
	*Penicillium brevicompactum*	1	0.473	0.204
	*Penicillium corylophilum*	1	0.374	0.079
	*Penicillium variabile*	1	0.415	0.172

(4)	*Penicillium purpurogenum*	1	1.000	0.028

(5)	*Acremonium strictum*	2	0.543	0.109
	*Alternaria alternata*	2	0.576	0.137
	*Cladosporium cladosporioides *(Type 1)	2	0.755	0.310
	*Cladosporium cladosporioides *(Type 2)	2	0.478	0.229
	*Cladosporium herbarum*	2	0.507	0.121
	*Epicoccum nigrum*	2	0.648	0.349

(6)	*Aspergillus flavus*	1	0.299	0.013
	*Aspergillus niger*	1	0.429	0.090
	*Aspergillus sydowii*	1	0.375	0.059
	*Aspergillus unguis*	1	0.499	0.087
	*Penicillium spinulosum*	1	0.145	0.030
	*Aspergillus ustus*	2	0.433	0.127
	*Penicillium chrysogenum *(Type 2)	2	0.429	0.133

(7)	*Aureobasidium pullulans*	1	0.326	0.177
	*Eurotium amstelodami*	1	0.538	0.283
	*Scopulariopsis brevicaulis*	1	0.547	0.173
	*Stachybotrys chartarum*	1	0.429	0.163
	*Mucor racemosus*	2	0.469	0.179
	*Rhizopus stolonifer*	2	0.348	0.060
